# Utilizing artificial intelligence to optimize psychological trauma intervention in social assistances

**DOI:** 10.3389/fpsyg.2026.1578545

**Published:** 2026-04-21

**Authors:** Yu Zhang, Wenzuixiong Xiong, Yuxiang Gao

**Affiliations:** 1 School of Law and Economics, Wuhan University of Science and Technology, Wuhan, China; 2 School of Art, Hubei University, Wuhan, China

**Keywords:** artificial intelligence, intelligent auxiliary, diagnostic system, privacy protection, psychological trauma, social assistance

## Abstract

**Introduction:**

Psychological trauma is prevalent among beneficiaries of social assistance, posing significant challenges to effective intervention. Traditional psychological support methods are often constrained by limited resources, delayed responses, and insufficient personalization. Against this backdrop, artificial intelligence (AI) offers new opportunities to enhance the efficiency and precision of psychological trauma interventions. This study explores the feasibility and effectiveness of integrating AI technologies into social assistance systems.

**Methods:**

This study adopts a mixed analytical approach. First, it reviews the current applications of AI in mental health, focusing on natural language processing, emotion recognition, and personalized recommendation systems. Based on this, an AI-driven framework for psychological trauma intervention is proposed, including the development of intelligent auxiliary diagnostic systems and personalized intervention plans. Empirical data are collected through questionnaire surveys to evaluate user experiences and intervention outcomes.

**Results:**

The findings indicate that AI-assisted interventions significantly improve user satisfaction and mental health outcomes. Specifically, 75% of respondents report being satisfied or very satisfied with the intelligent mental health system. Approximately 60% of participants experience noticeable improvements in their mental health status. Furthermore, 85% of respondents consider the personalized intervention plans to be well-aligned with their individual needs and circumstances.

**Discussion:**

The results demonstrate that AI technologies can effectively address key limitations of traditional psychological interventions by enhancing accessibility, responsiveness, and personalization. The integration of AI into social assistance systems not only improves intervention outcomes but also offers scalable solutions for resource-constrained settings. This study provides empirical support for the application of AI in social assistance and highlights its transformative potential in advancing mental health governance.

## 1 Introduction

In the context of the new era, social assistance is an important social activity. It aims at providing necessary support and resources to vulnerable groups to improve their quality of life and social integration (Hou et al., 2020; Mishra, 2020; Shao et al., 2020). However, in this process, people often overlook an important issue: beneficiaries may face various psychological traumas, posing challenges to assistance work. Psychological traumas, such as post-traumatic stress disorder (PTSD), anxiety, and depression. They affect the mental health of beneficiaries and may hinder their social integration and the process of rebuilding their lives (Jolly et al., 2020). Traditional psychological intervention methods, such as counseling and therapy, although can alleviate psychological traumas to some extent, are plagued by many issues. Resource scarcity, insufficient services, and lack of professional personnel manpower result in many beneficiaries not receiving timely and effective help. Especially for vulnerable groups in social assistance, due to the uniqueness of their experiences and the diversity of their needs, traditional psychological intervention methods often prove inadequate (Alam, 2022; Fu et al., 2020; Zhang et al., 2020).

With the continuous advancement of technology, artificial intelligence (AI) technology has gradually become a new choice for optimizing psychological trauma intervention in social assistance (Sun et al., 2020). Natural language processing (NLP) technology can analyze the linguistic expressions of beneficiaries. Emotion recognition technology can identify changes in beneficiaries’ emotions. Personalized recommendation technology can recommend corresponding intervention plans based on the characteristics of beneficiaries. The application of these technologies provides new avenues and possibilities for improving mental health (Torous et al., 2021; Xu et al., 2021). When services are provided solely by humans, issues such as uneven resource distribution, shortage of professionals, and slow response time may arise. This is especially problematic in remote areas or during emergencies, where mental health services often struggle to meet demand in a timely and effective manner.

This work aims to explore the feasibility and effectiveness of utilizing AI technology to optimize psychological trauma intervention in social assistance. First, this work analyzes the current situation of psychological trauma in social assistance and discusses the limitations of traditional psychological intervention methods. Next, this work introduces the current application status and potential advantages of AI technology in the field of mental health. Then, it proposes methods and strategies to optimize psychological trauma intervention in social assistance using AI technology. Finally, this work looks forward to the future prospects of AI in the field of social assistance and emphasizes the importance of addressing ethical and privacy protection issues while advancing technology. This work is expected to provide new ideas and methods to improve the mental health of beneficiaries and contribute to the further development and improvement of social assistance work.

## 2 Literature review

In the field of mental health, the application of AI technology has become increasingly widespread. NLP techniques can analyze the speech of beneficiaries, emotion recognition technology can identify changes in the emotions of beneficiaries, and personalized recommendation technology can recommend appropriate psychological intervention programs based on the characteristics of the beneficiaries. The application of these technologies provides new avenues and possibilities for improving mental health (Rowe and Lester, 2020). For example, Zhang et al. (2022) utilized emotion recognition technology to analyze the speech and behavior of social assistance recipients, timely identifying and intervening in the development trends of psychological problems, achieving certain effectiveness. Additionally, Goldberg et al. (2020) studied an NLP-based method for analyzing psychological counseling dialogues, providing counselors with better tools to understand the needs of beneficiaries.

Cognitive Behavioral Therapy (CBT) is a widely used psychological treatment method aimed at improving mental health by changing an individual’s thought patterns and behavioral habits. The core assumption of CBT is that an individual’s thoughts and beliefs directly influence their emotions and behaviors. By identifying and challenging negative thought patterns, individuals can learn more positive and adaptive coping strategies. Affective computing is a field of study focused on how to enable computers to understand and generate emotions. By analyzing users’ behavior, speech, and physiological signals, affective computing can recognize and understand users’ emotional states, providing more personalized support and services. AI systems can leverage technologies such as speech recognition, facial expression recognition, and physiological signal monitoring to accurately identify changes in users’ emotions. These technologies can help the system promptly detect emotional issues and offer appropriate support. As AI technology continues to advance and develop, more and more studies are beginning to explore the possibility and effectiveness of utilizing AI technology to optimize psychological trauma intervention in social assistance. For instance, Parker and Grote (2019) designed intervention programs targeting different types of psychological trauma using personalized recommendation technology, achieving good results. These studies suggest that AI technology provides new ideas and methods for optimizing psychological trauma intervention. Leung et al. (2021) explored the application of AI-based virtual psychotherapy in their research. They developed a virtual psychotherapy system that analyzed users’ speech and emotions through NLP techniques, and provided personalized psychological support and advice based on the analysis results. The research results indicate that the system effectively alleviates users’ anxiety and depression symptoms, improving their mental health levels. This study provides new ideas and methods for using AI technology in psychotherapy.

Mylona et al. (2022) primarily focused on the analysis of psychological counseling dialogues based on NLP technology. They analyzed the speech expressions of beneficiaries and counselors in psychological counseling dialogues and studied the interaction patterns and influencing factors between the two. The study found that the linguistic features in psychological counseling dialogues were closely related to the psychological state of the beneficiaries and could be used to predict and assess their mental health levels. This study provides counselors with better tools to understand the needs of beneficiaries, thus helping to improve the effectiveness and quality of psychological counselling. Barbalat et al. (2024) concentrated on designing psychological intervention plans using personalized recommendation technology. They developed a personalized intervention system based on machine learning algorithms, recommending corresponding psychological intervention plans based on the characteristics and needs of the beneficiaries. The research results showed that personalized intervention plans are more effective in alleviating psychological trauma and improving mental health compared to traditional generic intervention plans. This study provides new ideas and methods for improving the specificity and effectiveness of psychological intervention. Fei et al. (2020) aimed to monitor mental health conditions using emotion recognition technology. They developed an emotion recognition system that identified users’ emotional states by analyzing their speech and behavior and provided corresponding psychological support and advice based on emotional changes. The research results demonstrated that the system accurately identified users’ emotional changes and effectively improved their mental health levels. This study provides new avenues and possibilities for using emotion recognition technology for mental health monitoring and intervention. Emotional changes are a crucial basis for AI systems to provide support and recommendations. The system uses NLP and emotion recognition technologies to conduct a comprehensive analysis of the beneficiary’s voice, facial expressions, and physiological indicators to accurately identify their emotional state and mental health condition. Specifically, the system looks for emotional vocabulary, tone variations in the voice, microexpressions, and muscle activity in the face, and monitors physiological indicators such as heart rate and blood pressure to comprehensively assess emotional changes. These emotional changes are calculated using algorithmic models, which quantify the emotional state based on the beneficiary’s historical and real-time data, providing a foundation for subsequent support and recommendations.

The research conducted by these scholars provides crucial theoretical and practical support for the application of AI technology in the field of mental health. Their work enables a better understanding and utilization of AI technology, providing new ideas and methods for improving mental health. In the future, people can further draw on their research findings to continuously improve related technologies and methods, making greater contributions to the development of the field of mental health. Moreover, with the continuous progress and application of AI technology, there is the opportunity to delve deeper into research, explore further, continuously refine relevant technologies and methods, and make greater contributions to enhancing the mental health of beneficiaries. However, the process of utilizing AI technology for psychological intervention also needs to pay attention to ethical and privacy protection issues to ensure the benign application of technology, and truly benefit the recipients of social assistance.

A review of relevant literature demonstrates that AI technology provides new ideas and methods for psychological trauma intervention in social assistance, carrying significant theoretical and practical implications.

## 3 Methods

### 3.1 Theoretical analysis of psychological trauma in social assistance

Social assistance aims to provide necessary support and resources to individuals in need, to improve their quality of life and social integration. However, individuals receiving social assistance often experience various misfortunes and pressures, which may lead to the emergence of psychological trauma. Theoretical analysis of psychological trauma in social assistance helps to deepen understanding of its mechanisms and influencing factors, providing a theoretical basis and guidance for psychological intervention (Boyer, 2022; Qiu and Wu, 2024; Saiyu et al., 2022; Mitchell, 2019). When discussing the concept and characteristics of psychological trauma in social assistance centers, it is important to clearly distinguish between the traumatic events themselves and the process of receiving social assistance. In fact, most clients, after experiencing traumatic events (such as natural disasters, violence, abuse, and loss of loved ones), actively or passively seek social assistance services to obtain necessary support, aid, and recovery. These traumatic events are the direct causes of the client’s psychological trauma, not the process of receiving social assistance itself. In some cases, clients may encounter issues such as communication barriers, inadequate services, and lack of trust while receiving social assistance. While these issues are not directly equivalent to the traumatic events themselves, they can negatively impact the client’s psychological state and exacerbate their trauma. Therefore, when providing social assistance services, it is essential to focus on the traumatic events themselves and on the potential problems in the service process. This can ensure that clients receive comprehensive and effective psychological support.

1. Concept and Characteristics of Psychological Trauma in Social Assistance: Psychological trauma in social assistance refers to the phenomenon of psychological trauma occurring during the process of receiving social assistance. This trauma is usually caused by individuals experiencing a series of negative events or hardships, such as domestic violence, natural disasters, and unemployment, leading to psychological distress. Social assistance recipients often find themselves in disadvantaged positions, lacking social support and resources, making them more susceptible to the influence of external events and thus experiencing psychological trauma.
2. Types and Manifestations of Psychological Trauma: In the context of social assistance, psychological trauma can manifest in various types, including PTSD, anxiety, and depression. PTSD is a common type of psychological trauma, characterized by persistent negative emotions and behavioral reactions triggered by traumatic events. Anxiety and depression are also common emotional issues, characterized by sustained feelings of tension, fear, and despondency. These types of psychological trauma are prevalent among social assistance recipients, severely impacting their mental health and quality of life.
3. Mechanisms and Influencing Factors of Psychological Trauma: The occurrence mechanism of psychological trauma in social assistance is a complex process influenced by individual, environmental, and societal factors. Individual factors include mental health status and coping strategies; environmental factors encompass family environment and social support; and societal factors involve socioeconomic status and cultural background. These factors interact with each other, collectively affecting the individual’s risk and severity of psychological trauma. Psychological trauma is often closely related to the events individuals experience, such as accidents, natural disasters, wars, and violent incidents. The suddenness, severity, and unpredictability of these events can cause immense psychological shock to individuals, leading to trauma. Factors such as an individual’s psychological resilience, coping mechanisms, and personality traits play significant roles in the occurrence of psychological trauma (Mitchell, 2019). The more fragile, vulnerable, or lacking effective coping strategies an individual is, the more susceptible they are to the effects of psychological trauma. The adequacy of social support systems plays a crucial role in the occurrence and impact of psychological trauma. Lack of social support, adverse environments, social conflicts, and other factors exacerbate the severity of individual psychological trauma. An individual’s cognition and interpretation of events also affect the occurrence of psychological trauma. For example, different interpretations of the same event may result in varying degrees of trauma, and positive cognitive approaches can alleviate the severity of trauma. Psychological trauma is not merely a psychological state but also affects the individual’s physiological functions. Prolonged psychological trauma may lead to a series of physical problems, such as insomnia, headaches, and digestive issues, thereby affecting the individual’s quality of life (Zhang et al., 2020).

### 3.2 The use of AI in the field of mental health

Traditional psychological intervention methods, such as counseling and therapy, although to some extent alleviate psychological trauma, are fraught with various issues. For instance, resource scarcity, insufficient services, and a shortage of professional manpower often result in many recipients not receiving timely and effective assistance (Sanderson et al., 2020; Sloshower et al., 2019). However, with the rapid development and continuous innovation of AI technology, its application in the field of mental health is becoming increasingly widespread. It offers new avenues and methods for the diagnosis, intervention, and treatment of mental health issues. AI technology, with its powerful data processing and analysis capabilities, has shown tremendous potential in the field of mental health, providing psychologists, doctors, and patients with more intelligent and personalized services (Alowais et al., 2023; Doraiswamy et al., 2020; Lee and Yoon, 2021).

**Figure 1:**
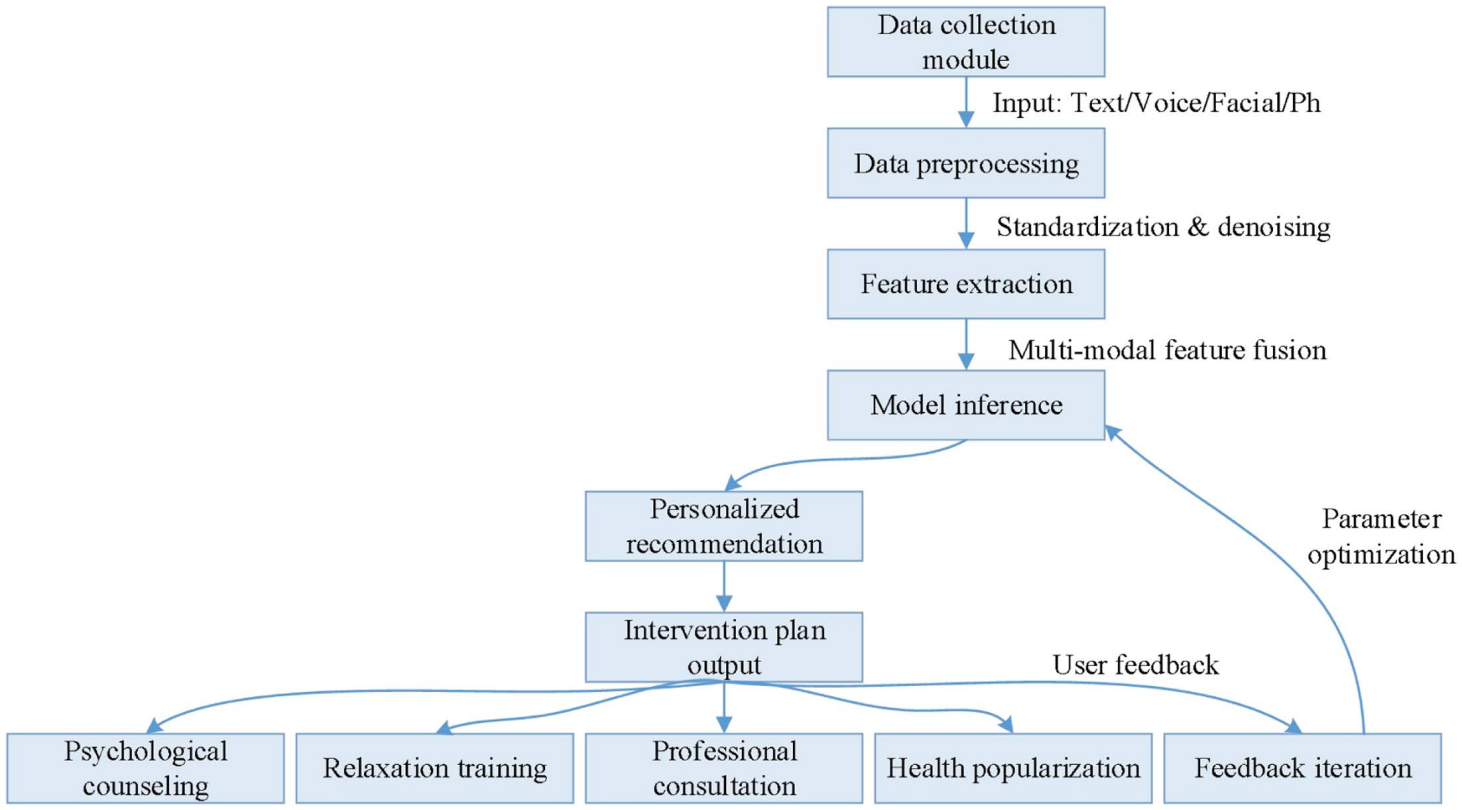
IMHAS algorithm flow chart.

**Figure 2:**
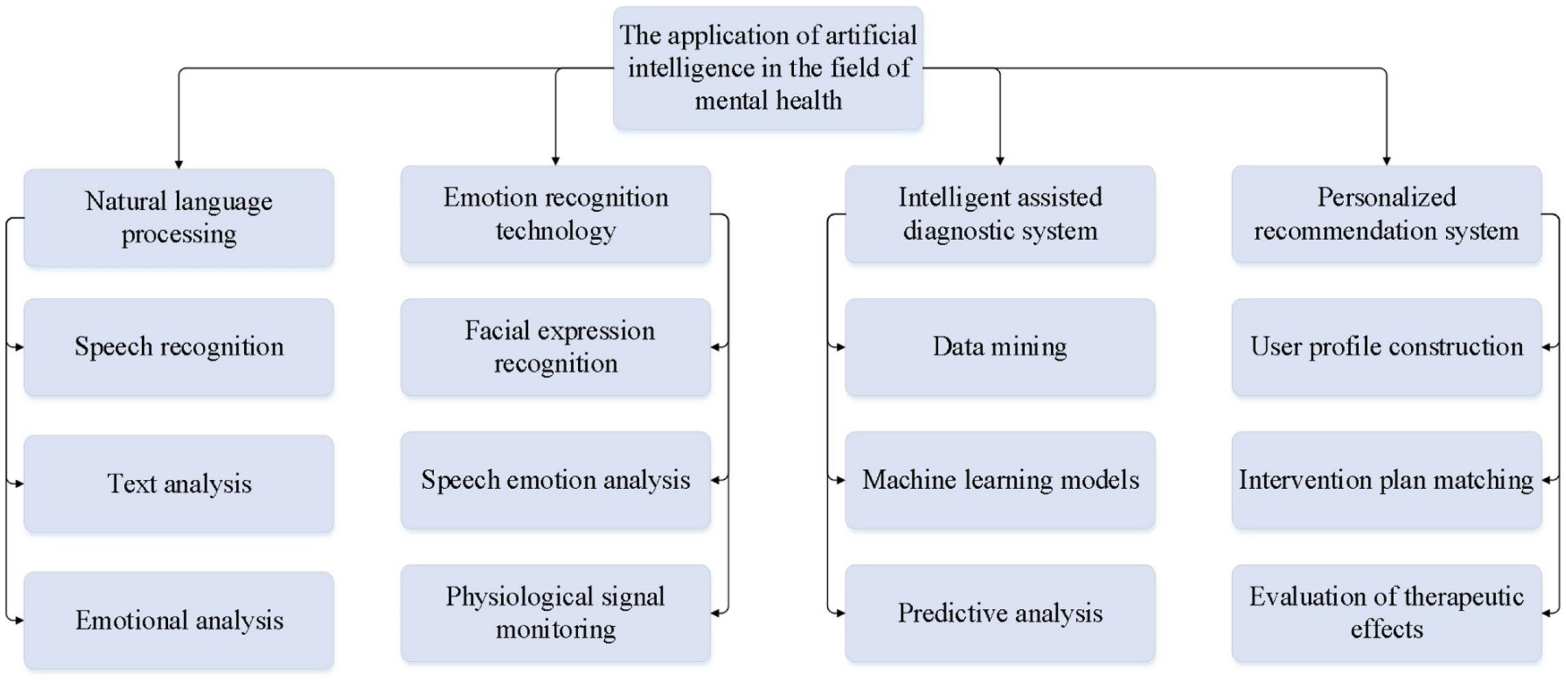
The application of AI in the field of mental health.

The Intelligent Mental Health Assistance System (IMHAS) is an integrated platform that combines NLP, emotion recognition, personalized recommendation, and data mining technologies. The flow chart of IMHAS algorithm is shown in Figure 1. The system is designed to provide personalized mental health support and intervention plans for individuals in need of social assistance. IMHAS consists of the following main modules. (1) User Interface Module: Users can access the system through various channels such as web portals, mobile apps, and WeChat mini-programs. The system records users’ personal information and usage history. (2) Data Collection Module: The system collects data from users via text input boxes, voice input, and camera-captured facial expressions. It also gathers physiological signal data through wearable devices. (3) Data Processing Module: The system employs deep learning models like BERT and Transformer for sentiment analysis, semantic understanding, and keyword extraction of users’ text input. It uses the Google Speech-to-Text API or other speech recognition engines to convert voice input into text. It also adopts OpenCV along with deep learning models to recognize users’ facial expressions. (4) Emotion Recognition Module: By integrating the results of NLP, speech recognition, and facial expression analysis, the system comprehensively evaluates users’ emotional states, monitors real-time changes in their emotions, and records historical emotional data. (5) Personalized Recommendation Module: Based on users’ personal information, mental health status, and historical data, the system constructs users’ profiles and recommends relevant mental health articles, videos, and exercises according to users’ interests and needs. (6) Data Storage and Management Module: The system encrypts user data using algorithms like AES to ensure data security. (7) Assessment and Feedback Module: The system periodically sends satisfaction surveys to users, collects feedback, evaluates users’ mental health using mental health scales, and tracks changes before and after interventions. Figure 2 illustrates the application of AI in the field of mental health.

1. NLP Technology: The application of NLP technology in the field of mental health is becoming increasingly widespread. By analyzing the speech expressions of beneficiaries, NLP technology assists psychologists and doctors in better understanding patients’ inner worlds and emotional states, thus providing more personalized and effective psychological support and intervention. For example, by analyzing the content and emotional tone of patients’ speech, NLP technology can automatically generate psychological assessment reports and intervention plans, providing scientific evidence for the diagnosis and treatment of mental health issues (Le Glaz et al., 2021). This work employs deep learning-based NLP models, such as BERT and Transformer, to perform sentiment analysis and semantic understanding of users’ text input. These models can identify users’ emotional states, extract keywords, and detect underlying themes, enabling more accurate psychological assessments.
2. Emotion Recognition Technology: It is another crucial application of AI technology in the field of mental health. By analyzing patients’ speech, facial expressions, and physiological indicators, emotion recognition technology can accurately identify patients’ emotional states and mental health conditions, providing timely and effective support for psychological intervention and treatment (Gual-Montolio et al., 2022). For example, emotion recognition technology can monitor changes in patients’ emotions, and promptly identify and intervene in the development trends of mental health issues, thereby improving the accuracy of prediction and diagnosis of mental health problems. This work utilizes speech recognition, facial expression recognition, and physiological signal monitoring (such as heart rate and skin conductivity) to comprehensively assess changes in users’ emotions. These technologies are trained using machine learning algorithms, enabling accurate identification of users’ emotional states across different contexts.
3. Intelligent-assisted Diagnosis System: The intelligent-assisted diagnosis system is another important application of AI technology in the field of mental health. By utilizing machine learning and data mining techniques to analyze a large amount of mental health data, this system can assist doctors in diagnosing various mental health problems quickly and accurately, and provide personalized treatment plans for patients (Chen et al., 2021). For example, it can predict patients’ disease risks and treatment outcomes based on their symptoms and mental health history, providing scientific evidence for clinical decision-making.
4. Personalized Recommendation System: The personalized recommendation system is another crucial application of AI technology in the field of mental health. By analyzing patients’ personal characteristics and health needs, this system can provide personalized mental health services and treatment plans for patients, thereby improving treatment effectiveness and patient satisfaction. Individuals of different age groups exhibit variations in their physiological, psychological, and social needs. Younger individuals may be more focused on physical fitness and mental health, while older adults may prioritize disease prevention and rehabilitation care. Gender differences are also significant in health needs. Women may be more concerned with breast health, prenatal care, and menopause management, whereas men may focus more on prostate health, cardiovascular health, and similar areas. The machine algorithm provides targeted health recommendations based on these gender differences. An individual’s lifestyle habits play a crucial role in their health. Unhealthy behaviors, such as smoking, alcohol consumption, and staying up late, increase the risk of illness. The machine algorithm offers health improvement suggestions based on these lifestyle habits and encourages the adoption of healthier living practices. The system employs collaborative filtering and deep learning algorithms to recommend personalized psychological intervention plans based on users’ personal characteristics and historical data. The recommendation system also continuously optimizes its suggestions based on user feedback.

### 3.3 Methods and strategies for optimizing psychological trauma intervention in social assistance centers using AI

The effectiveness and quality of psychological trauma intervention in social assistance centers are directly related to the mental health and social integration of vulnerable groups. Traditional psychological intervention methods have various limitations, while the application of AI technology provides new ideas and methods for optimizing psychological trauma intervention in social assistance centers. This section explores methods and strategies for optimizing psychological trauma intervention in social assistance centers using AI technology.

**Figure 3:**
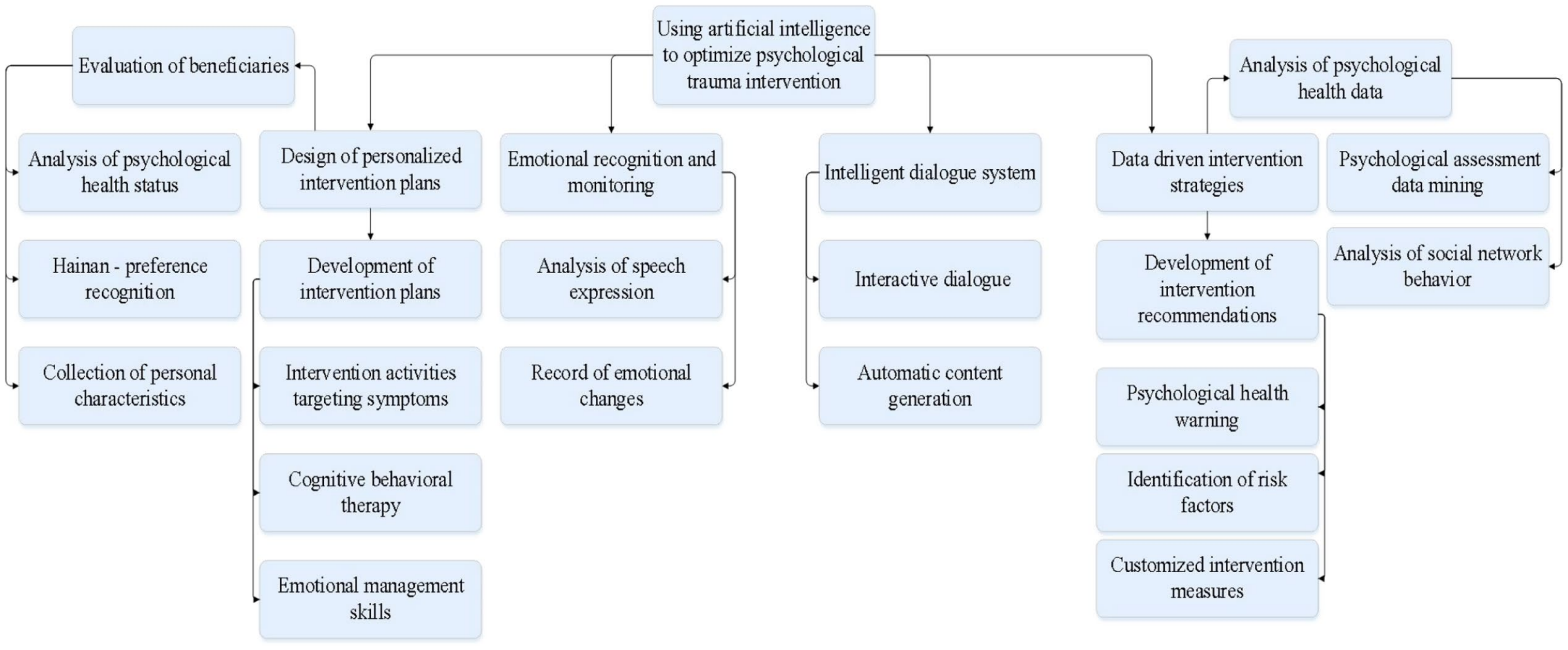
Methods and strategies for optimizing psychological trauma intervention using AI.

In a certain social assistance institution, to better help beneficiaries cope with mental health issues, they have introduced an IMHAS intelligent mental health assistance system. This system, based on AI technology, combines NLP, emotion recognition, and intelligent dialogue technologies to provide personalized psychological support and intervention for beneficiaries. The process of system evaluation involves three main steps: data preprocessing, feature extraction, and model prediction. First, the system preprocesses the raw data from beneficiaries, including voice, facial expressions, and physiological indicators, through operations such as noise reduction, filtering, and normalization. These steps improve the quality and accuracy of the data. Next, the system extracts feature related to emotional changes, such as the frequency of emotional vocabulary, amplitude of tone variation, and key points in facial expressions. These features are used in subsequent model predictions. Finally, the extracted features are fed into a trained algorithm model. The model predicts and assesses the emotional state of the beneficiary based on these features, resulting in a quantified evaluation of emotional changes. Figure 3 illustrates the methods and strategies for optimizing psychological trauma intervention using AI.

When constructing the AI health management system, particular attention is paid to matching individual preferences with appropriate intervention measures. Besides, corresponding algorithms are designed to ensure that the system can accurately recommend interventions tailored to clients’ needs. First, a user profiling approach is adopted, wherein personal information, health data, and historical behavior records are collected and analyzed to create a personalized profile for each client. This profile includes various dimensions of information such as the client’s health status, disease risk, lifestyle habits, and interests, providing a foundation for matching subsequent intervention measures. Next, a comprehensive intervention measure database is established, containing a wide range of interventions for different health issues and needs. These measures include, but are not limited to, medication treatments, physical therapy, psychological interventions, nutritional guidance, and exercise recommendations. This database is not a static, pre-loaded list but is continuously updated and refined based on the latest medical research and practical experience.

During the matching process, the system selects the interventions that best align with the client’s needs and preferences from the intervention database, based on a multi-factor comprehensive evaluation. This matching process is based on a multi-factor comprehensive evaluation, which includes, but is not limited to, the client’s health status, disease risk, lifestyle habits, interests, and specific health goals and expectations.

Finally, the AI system delivers these intervention measures to clients through multiple channels, such as mobile apps and smart wearable devices, providing real-time, personalized health suggestions and interventions. The system also offers remote medical consultations and expert advice services to help clients better understand and implement the recommended interventions. When selecting intervention measures, multiple factors are considered, including the effectiveness, safety, feasibility, and client acceptance of the measures. Through iterative refinement and optimization, the system gradually improves the accuracy and satisfaction of the intervention matches.

1. Personalized Intervention Plan Design: This system first conducts personalized assessments of beneficiaries, collecting information such as their personal characteristics, mental health status, and preferences. Based on the beneficiary’s emotional changes and mental health status, the AI system provides personalized support and recommendations. These supports and suggestions mainly include encouragement, comfort, and psychological counseling, aimed at helping beneficiaries alleviate negative emotions such as anxiety and depression, while improving their overall mental health. The system automatically selects the most suitable support and suggestions to deliver based on the beneficiary’s emotional state and needs. For example, when the system detects a significant level of anxiety in the beneficiary, it will automatically generate and push encouraging and supportive messages. Similarly, when the system identifies a need for psychological counseling, it will recommend professional counselors or psychological intervention resources for the beneficiary to choose from.
2. Emotion Recognition and Mood Monitoring: The system analyzes the speech expressions and emotional states of beneficiaries to monitor their emotional changes and mental health status in real-time. For example, when the system detects negative emotional vocabulary or tone in the beneficiary’s speech, it immediately alerts the psychological counselor for intervention. Meanwhile, the system also records the history of the beneficiary’s emotional changes, providing more comprehensive and accurate information support for psychological intervention.
3. Intelligent Dialogue System: Through intelligent dialogue technology, the system communicates and interacts with beneficiaries, providing personalized psychological support and advice. For example, the system can automatically generate corresponding intervention content based on the beneficiary’s speech expressions and emotional states, such as encouragement, comfort, and advice, to help beneficiaries understand and cope with mental health issues.
4. Data-Driven Intervention Strategies: By analyzing the mental health data and behavioral data of social assistance recipients, the system extracts valuable information and patterns to provide scientific evidence for psychological intervention. For example, the system can identify potential mental health issues and risk factors, and propose corresponding intervention recommendations and measures by analyzing the psychological assessment data and social network behavior data of beneficiaries.

The above examples demonstrate the application of AI technology in psychological trauma intervention in social assistance centers. By introducing intelligent assistance systems, social assistance institutions can provide beneficiaries with more personalized, timely, and effective psychological support and intervention, improving the targeting, effectiveness, and scientificity of psychological intervention. This system provides new ideas and methods for the improvement and enhancement of social assistance work.

### 3.4 Questionnaire survey

This work employs a variety of methods to comprehensively analyze the content and emotional tone of patent presentations, and monitor changes in patients’ emotions. First, patent presentation data relevant to this work are collected from multiple authoritative patent databases. These datasets cover patent presentations from different fields and time periods, ensuring the diversity and representativeness of the sample. Strict privacy protection and data security protocols are followed during data collection to ensure the legality and compliance of all data. To evaluate the intervention effect of IMHAS on psychological trauma, an independent-samples t-test is used to analyze the intergroup differences in Patient Health Questionnaire-9 (PHQ-9) and Generalized Anxiety Disorder-7 (GAD-7) scores before and after the intervention to examine the significance of differences in intervention effects between the two groups. A paired-samples t-test is used to analyze the intragroup differences in scores before and after the intervention in each group to examine the significance of the intervention effect within a single group.

To analyze the content and emotional tone of the patent presentations, NLP techniques are employed. Initially, text preprocessing steps such as tokenization and part-of-speech tagging are used to clean and standardize the patent presentations. Then, sentiment analysis algorithms are applied to quantify the emotional tendencies within the presentations, including positive, negative, and neutral emotions. Additionally, methods like keyword extraction and topic modeling are used to analyze the core content and key information of the patent presentations. To monitor changes in patients’ emotions, an emotion recognition system based on multimodal data from patient voice, facial expressions, and text communication is designed. This system can capture and analyze patients’ emotional changes in real-time and generate emotion reports, providing healthcare professionals with timely emotional monitoring and intervention support.

Conducting a questionnaire survey is a crucial means to evaluate the effectiveness and user satisfaction of the IMHAS in psychological trauma intervention in social assistance centers. When designing the questionnaire survey, several dimensions need to be considered:

1. Satisfaction Evaluation: it is to evaluate the overall satisfaction of the respondents with the IMHAS and the satisfaction with various functions, including the usability, practicality, and effectiveness of the system. Satisfaction primarily reflects the beneficiaries’ overall evaluation and user experience with the system, while effectiveness assesses the system’s impact on improving the beneficiaries’ mental health status. To measure client satisfaction with the AI system, a comprehensive customer satisfaction survey is designed. This questionnaire covers various aspects such as the system’s ease of use, user interface friendliness, response time, and level of personalization. It aims to gain a thorough understanding of the client’s feelings and experiences during system usage. When evaluating the effectiveness of the intervention measures, corresponding evaluation standards and indicators are established based on the client’s health status, disease type, and the specific content of the intervention. These standards and indicators may include improvements in physiological indicators (such as blood pressure and blood glucose levels), improvements in psychological states (such as anxiety and depression), and enhancements in quality of life.
2. Intervention Effect Assessment: This is to evaluate the effectiveness of the IMHAS in intervening in psychological trauma. This includes assessing whether the respondents feel an improvement in their mental health status and whether they find it easier to cope with difficulties and challenges.
3. Personalization Degree Evaluation: This is to evaluate the satisfaction of the respondents with the personalized intervention plans provided by the system. This includes assessing whether the system provides corresponding intervention plans based on individual characteristics and needs, and the specificity and practicality of the intervention plans.
4. Interaction Experience Evaluation: This is to evaluate the experience of the respondents with the system’s intelligent dialogue and emotion recognition functions. This includes assessing whether the interaction with the system is smooth, and whether the system can accurately recognize and understand the users’ emotional states.
5. Data Privacy Protection Assessment: This is to evaluate the system’s data privacy protection measures. This includes assessing whether the system can protect users’ personal privacy information and whether there are sufficient data security measures.

The participants in this study must meet the following criteria. They are aged between 18 and 65, voluntarily participate in the study, are capable of using a smartphone or computer, and agree to sign an informed consent form. Participants with severe cognitive impairments, mental illnesses, or those unable to cooperate in completing the study are excluded. All participants’ personal information is kept strictly confidential, and data are stored on encrypted servers, accessible only by authorized researchers. The system employs anonymization to ensure that users’ private information is not disclosed. A high priority is placed on protecting participants’ data privacy. Before recruiting participants, the purpose of the study, and the methods of data collection and use, are explained to them in detail; written consent is also obtained to ensure their voluntary participation after fully understanding the study’s content and risks. Two hundred social assistance recipients participate in the questionnaire survey. Two hundred questionnaires are distributed, 190 are collected, and after excluding invalid questionnaires, 180 valid questionnaires are obtained, with a recovery rate of 94.7%. To more comprehensively validate the effectiveness and advantages of the IMHAS in psychological health interventions, this work designs a control group with a total of 190 participants. One group receives services provided by the AI system, while the other group receives services from professional healthcare providers. The two groups are balanced in terms of age, gender, and health status to ensure fairness and accuracy in the comparison results.

The social assistance recipients vary significantly in age, educational level and digital literacy. If the model training data fails to fully cover all groups, algorithmic bias may arise. This study optimizes the sample structure through stratified sampling and adds a fairness-constrained loss function to model training to reduce the performance disparities among groups. Given that psychological interventions involve sensitive information such as users’ core emotional experiences and traumatic memories, any leakage of such information may cause secondary harm. In addition to adopting AES encryption for data storage, this study further implements strategies including the principle of data minimization, hierarchical access rights and data lifecycle management. In view of the possibility that some assistance recipients passively accept AI interventions due to factors such as the digital divide and psychological dependence, this study safeguards their right to independent choice through a dual-track intervention model. Users can switch between AI and human interventions at any time, and meanwhile improve their cognitive ability of technology through the AI psychological knowledge popularization module in the system, thus realizing technological empowerment.

Participants are recruited through various channels. First, recruitment information is posted in communities, medical institutions, and online platforms, clearly outlining the study’s purpose, requirements, participants’ rights, and protection measures. Next, collaborations are established with local medical institutions to invite eligible patients to participate. Finally, recruitment invitations are sent to potential participants via social media and email. To ensure the diversity and representativeness of the participants, clear recruitment criteria and screening processes are established. These criteria cover various aspects such as age, gender, and health status to ensure that the participants can accurately reflect the characteristics of the target population. During data collection, identical evaluation standards and questionnaires are used to objectively assess the service effects for both groups. These evaluation standards cover improvements in health indicators, service satisfaction, the effectiveness of intervention measures, and other aspects. Comparing the data from both groups allows for a clearer understanding of the differences and advantages between the AI system and human-provided services in health management.

## 4 Results

### 4.1 Basic information of survey participants

**Figure 4:**
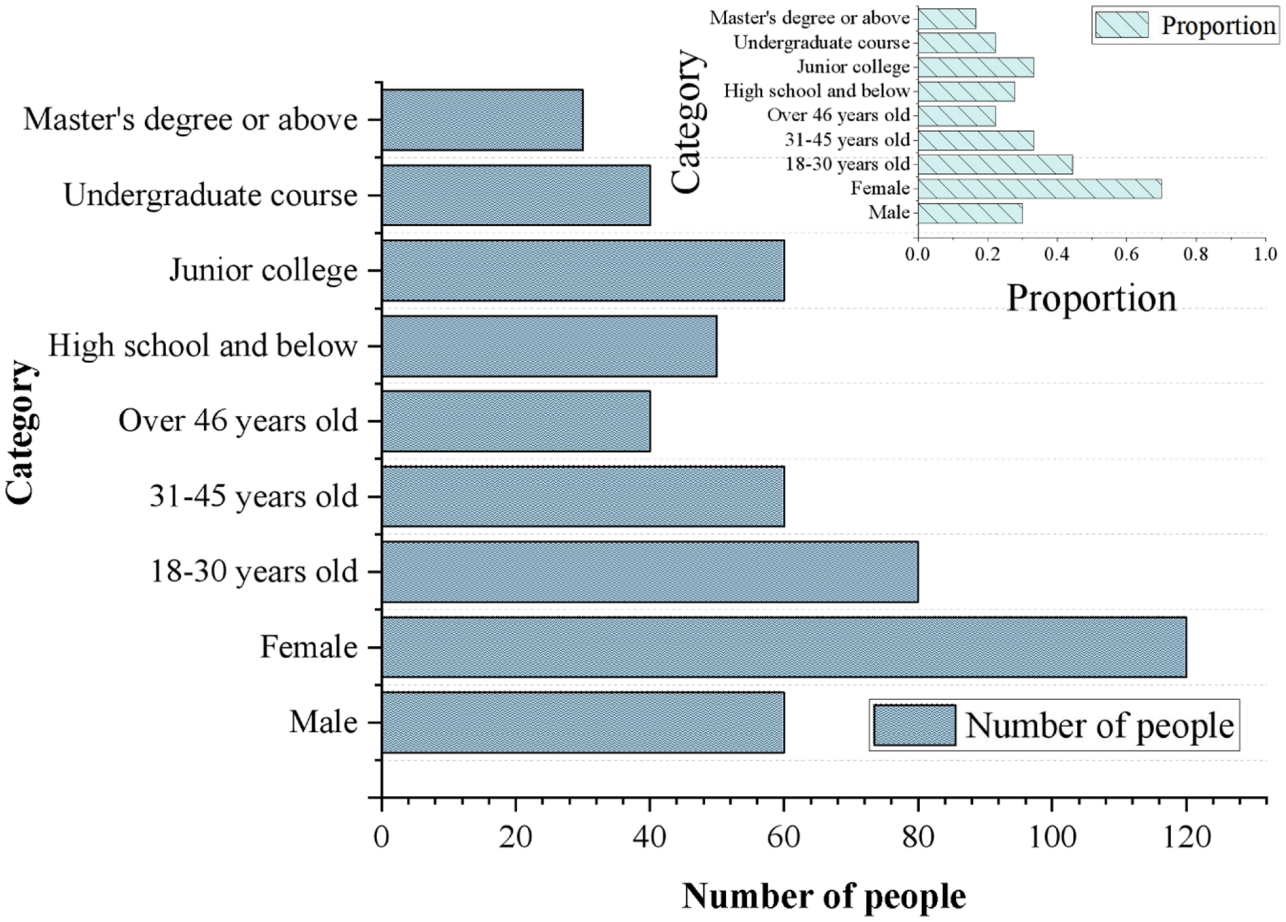
Basic information of survey participants.

Before assessing the satisfaction and intervention effects of the IMHAS, this work conducts statistical analysis on the gender, age group, and educational background of the survey participants. Figure 4 shows the basic information of the survey participants. Among the participants, the proportion of females is larger, accounting for 70%, while males make up 30%. This suggests that women are more proactive or willing to participate in the survey regarding the use of the system. The majority of participants are aged between 18 and 30, making up 44.45%. This likely reflects a growing awareness of mental health issues among young people, who tend to seek help through technological means. Participants aged 31 to 45 account for 33.33% of the total, indicating that individuals in this age group often face dual pressures from their careers and families. This potentially leads to a greater demand for mental health services. Participants aged 46 and above make up 22.22% of the total. It suggests that while this proportion is relatively small, individuals in this age group may have different needs and expectations when it comes to mental health services. Younger participants are typically more familiar with and confident in using new technologies, which results in higher comfort and satisfaction when using such systems. In contrast, older participants may experience lower comfort and satisfaction due to a lack of experience with technology or fear of new technologies. Regarding educational background, participants with a high school education or below account for 27.78%, suggesting that the IMHAS also reaches out to individuals with relatively lower levels of education. The highest proportion of participants holds an associate degree, accounting for 33.33%, indicating a potentially higher demand for mental health services among this educational group. Participants with a bachelor’s degree account for 22.22%, and they may have a better understanding of and demand for mental health services. Participants with a master’s degree or above constitute 16.67% of the total, and this group may have higher expectations for the quality of mental health services.

### 4.2 Satisfaction evaluation

**Figure 5:**
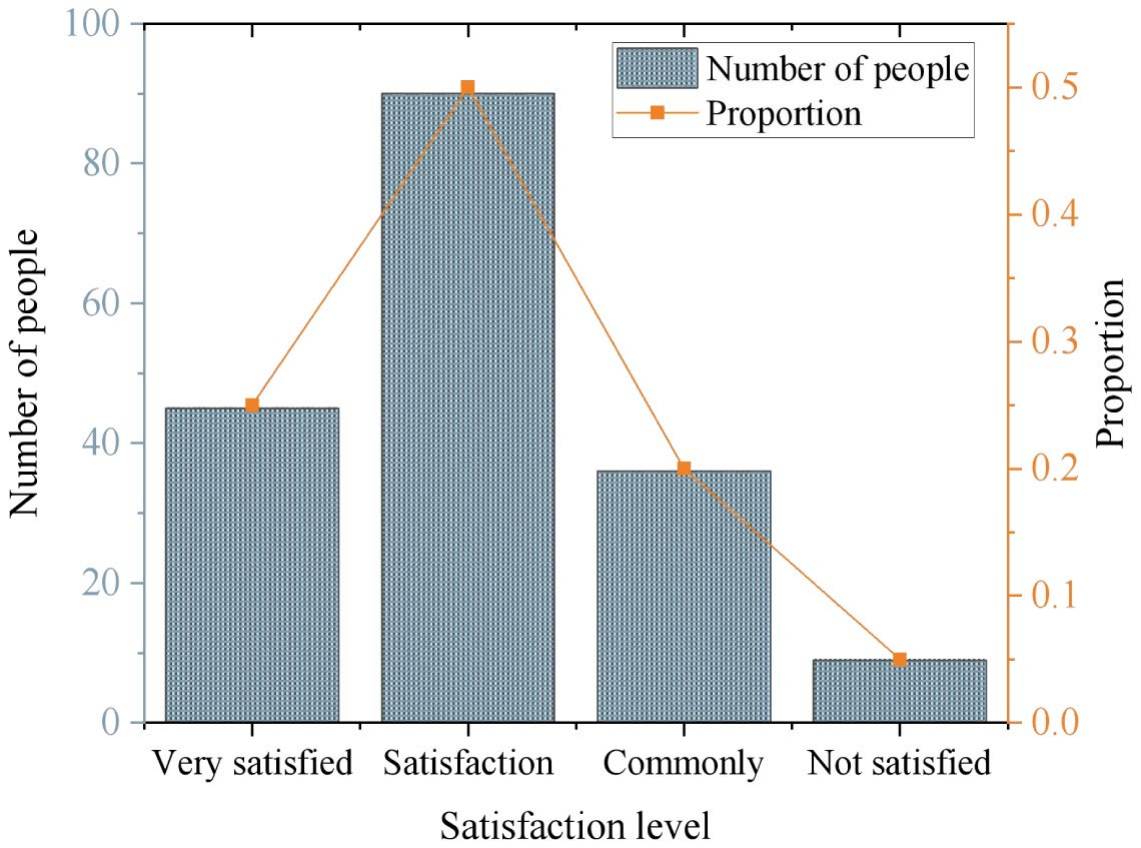
Distribution of users across different levels of satisfaction.

This work analyzes the satisfaction of the survey participants, with 75% of the respondents expressing satisfaction or high satisfaction with the IMHAS. 20% of the respondents indicate moderate satisfaction with the system, while 5% express dissatisfaction. Figure 5 illustrates the results. The respondents mainly express dissatisfaction with the system’s level of personalization, ease of use, and the effectiveness of the interventions. They feel that the system’s personalized recommendations are not accurate enough, the operational process is relatively cumbersome, and the interventions do not lead to significant improvements in their condition.

### 4.3 Intervention effect evaluation

**Figure 6:**
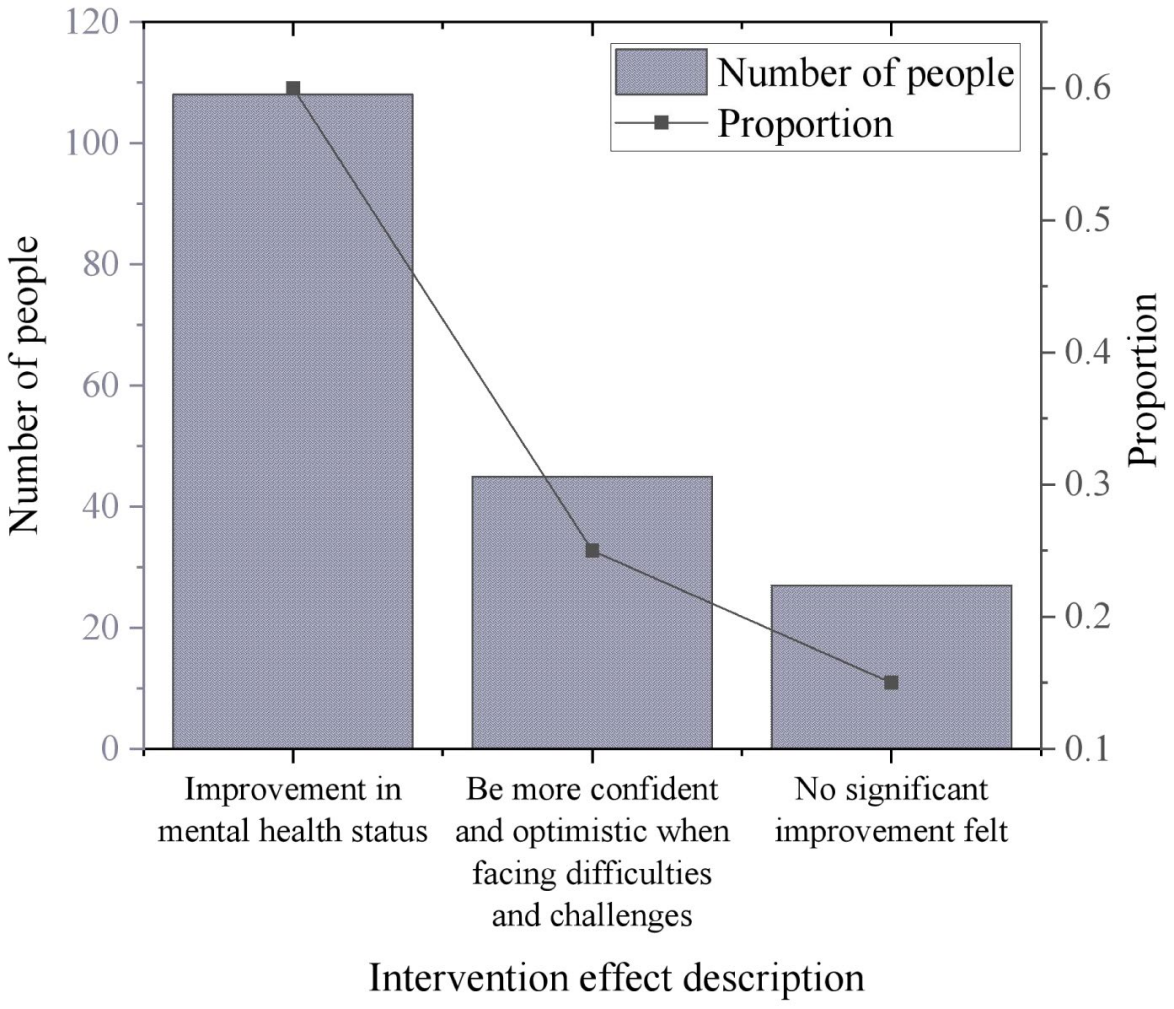
Impact of the IMHAS on users’ mental health improvement.

This work assesses the intervention effects on the survey participants. After using the system, 60% of the respondents report an improvement in their mental health condition. 25% of the respondents feel more confident and optimistic when facing difficulties and challenges. Through comparative analysis, it is found that respondents who demonstrate more confidence and optimism when facing challenges typically have simpler or more easily controllable symptoms. These individuals are more likely to accept and adapt to the interventions provided by the AI system, leading to quicker improvements. Some patients prefer face-to-face psychological counseling or treatment and are less inclined to undergo intervention through the AI system. For these individuals, even if the AI system offers personalized intervention plans, the effectiveness may be limited due to their lack of adaptation to or dislike for this approach. 15% of the respondents do not perceive a significant improvement in their mental health condition. Figure 6 illustrates the results.

### 4.4 Personalization assessment

**Figure 7:**
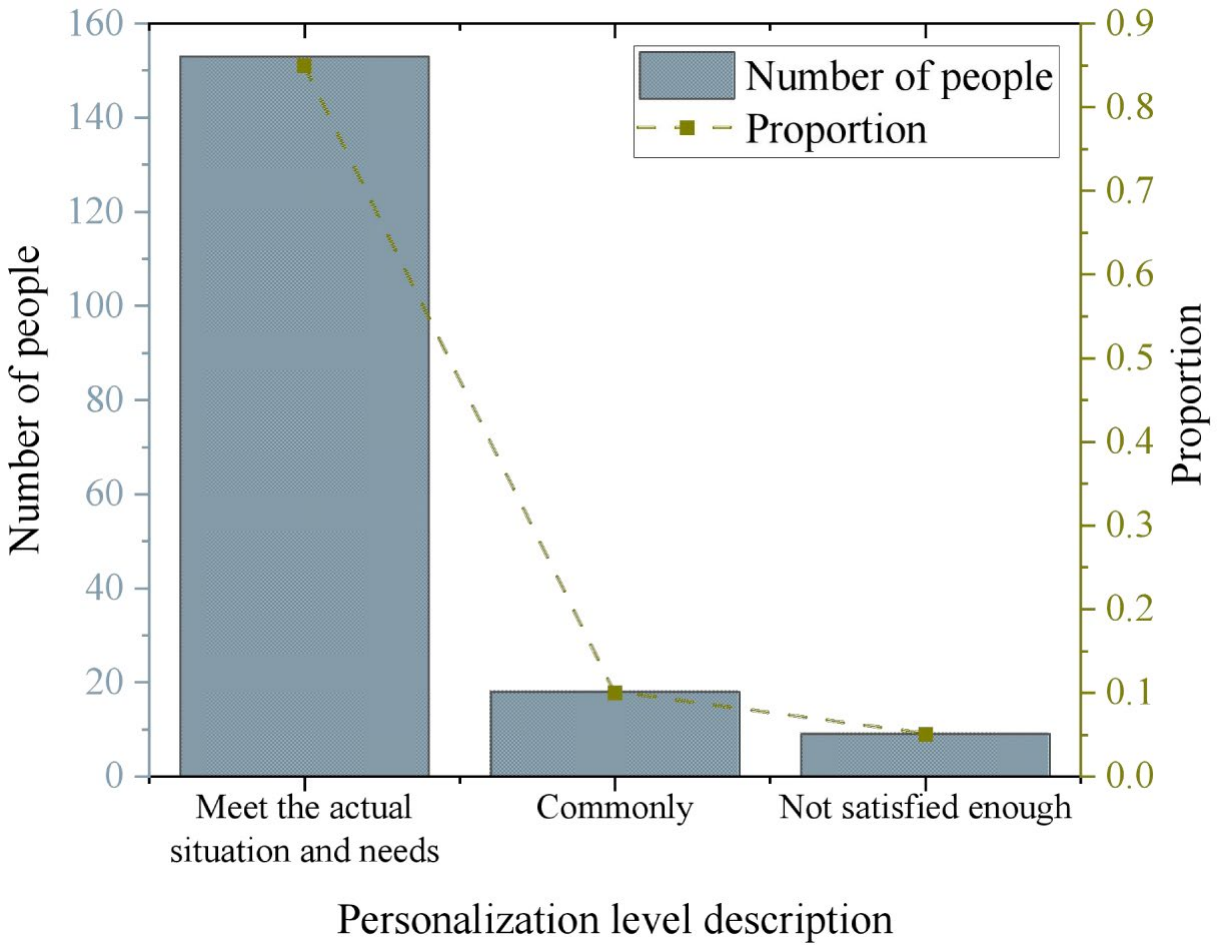
Users’ attitude towards personalized services provided by the IMHAS.

This work evaluates the level of personalization for the survey participants. 85% of the respondents believe that the personalized intervention plans provided by the system are quite suitable for their actual situations and needs. 10% of the respondents consider the personalized intervention plans provided by the system to be moderate. 5% of the respondents find the personalized intervention plans provided by the system unsatisfactory. Figure 7 illustrates the results.

### 4.5 Evaluation of interaction experience

**Figure 8:**
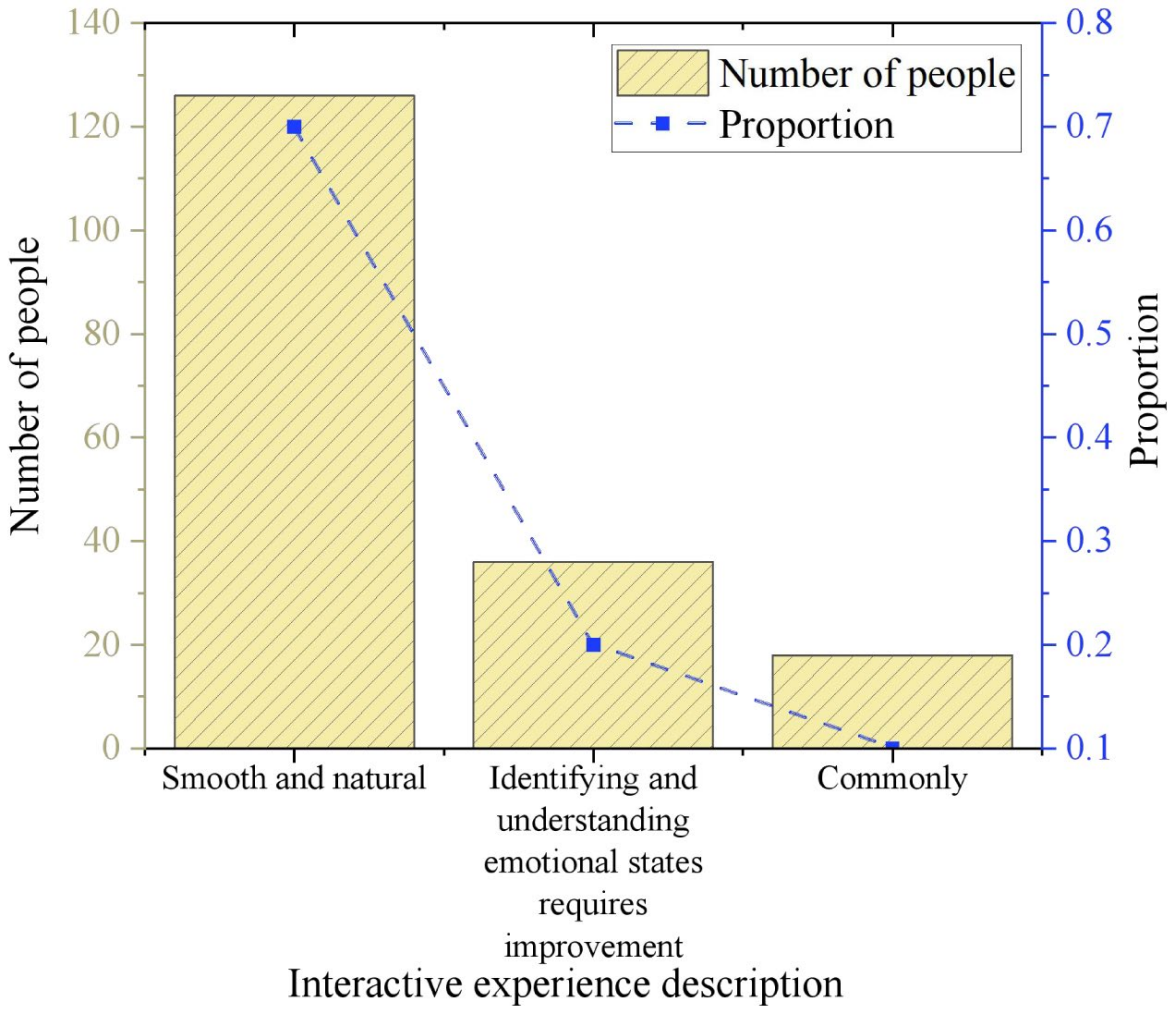
Users’ attitude towards interaction experience with the IMHAS.

This work evaluates the interaction experience of the survey participants. 70% of the respondents report that the interaction with the system is smooth and natural. 20% of the respondents indicate that there is room for improvement in the system’s recognition and understanding of user emotional states. 10% of the respondents rate their interaction experience with the system as average. Figure 8 displays the results.

### 4.6 Evaluation of data privacy protection

**Figure 9:**
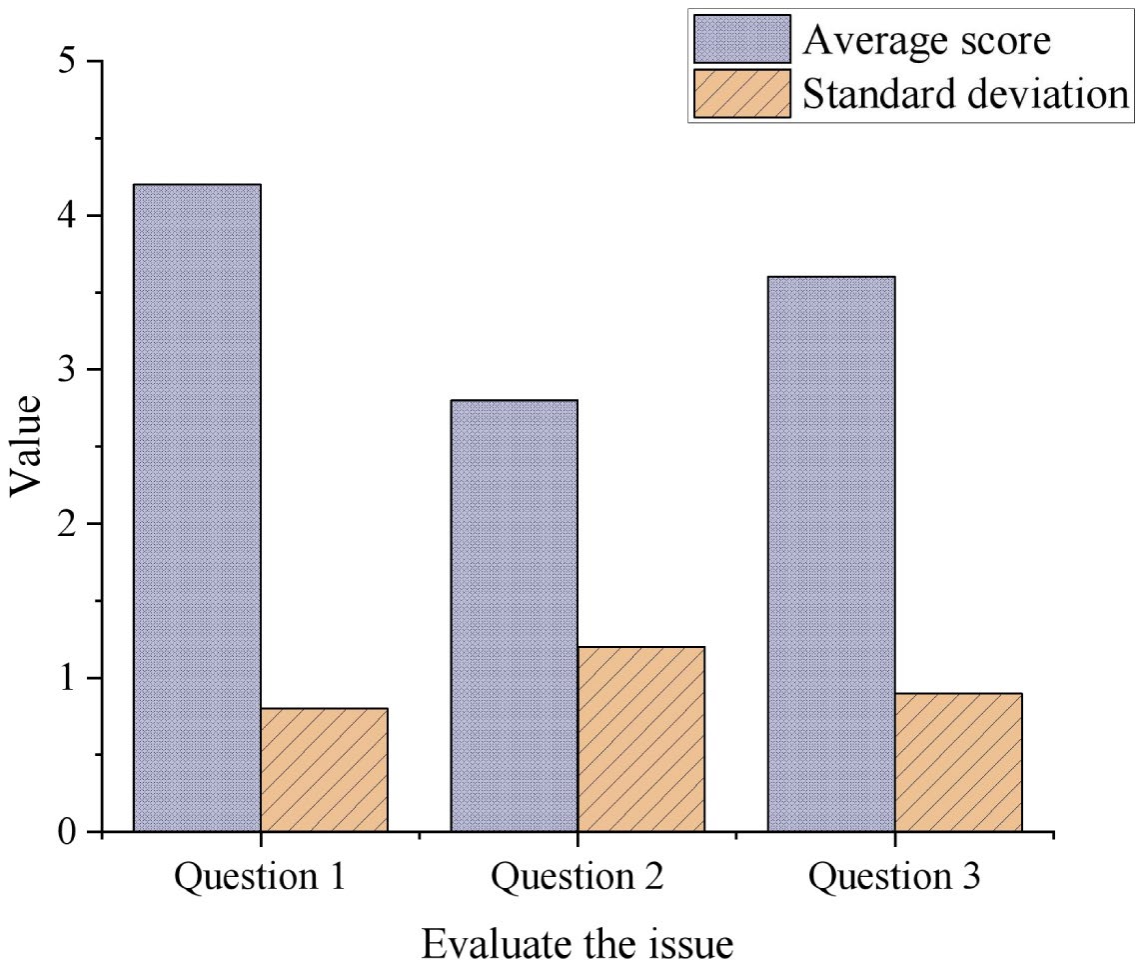
Statistical evaluation of data privacy protection.

This work assesses the survey participants’ perceptions of data privacy protection. Question 1: Do you believe the system effectively protects your personal privacy information? Average score: 4.2; Standard deviation: 0.8. Question 2: Are you concerned about your personal privacy information being leaked during the use of the system? Average score: 2.8; Standard deviation: 1.2. Question 3: Are you clear about how the system handles and protects your personal privacy information? Average score: 3.6; Standard deviation: 0.9. Figure 9 displays the results.

For questions 1 and 3, the respondents’ evaluations are relatively consistent, with data points clustered around the mean. It indicates that the respondents’ evaluations of the system’s ability to effectively protect personal privacy information and the clarity of the system’s handling and protection of personal privacy information are relatively stable. For question 2, the respondents’ evaluations are relatively dispersed, with data points spread over a wider range. It suggests that there is some variability in the level of concern among respondents about the potential leakage of personal privacy information during the use of the system, and the evaluations are not as consistent.

After the intervention, the mental health scale scores of the experimental group participants significantly increase, with noticeable reductions in anxiety and depression symptoms. A paired-samples t-test is adopted in this study to further compare the changes in PHQ-9 and GAD-7 scores before and after the intervention. The results show that the average PHQ-9 score of participants in the experimental group decreased by 4.5 points (SD = 1.2), and the average GAD-7 score decreased by 3.8 points (SD = 0.9). The paired-samples t-test confirms that these changes are statistically significant (PHQ-9: *t*(179) = −15.2, *p* < 0.001, Cohen’s *d* = 1.12; GAD-7: *t*(179) = −12.3, *p* < 0.001, Cohen’s *d* = 0.91). This indicates that the IMHAS system has a substantial effect on improving the mental health status of participants. The Shapiro–Wilk test is conducted, and the results showed that all continuous variables (PHQ-9 and GAD-7 scores) in both groups conformed to a normal distribution (*p* > 0.05), with no significant skewness or kurtosis anomalies. The Levene test verifies the homogeneity of variance for PHQ-9 and GAD-7 scores before and after the intervention in the two groups (*p* > 0.05), which meets the prerequisites for the independent-samples *t*-test and analysis of variance. In the mixed-design analysis of variance, the Mauchly’s test of sphericity is performed to verify the sphericity assumption, and the results show that *χ*^2^ = 2.31, df = 1, *p* = 0.129 > 0.05, indicating that the sphericity hypothesis is valid and no Greenhouse–Geisser correction is required.

Eighty-five percent of the experimental group participants believe that the personalized intervention plans provided by the IMHAS are very suitable for them, 10% think they are moderately suitable, and 5% think they are not suitable. Seventy percent of the participants in the control group believe that traditional psychological intervention plans suit their needs, 20% think they are moderately suitable, and 10% think they are not suitable. The experimental group shows significantly better applicability of personalized intervention plans compared to the control group (*p* < 0.05).

### 4.7 Psychological intervention strategies

Analyzing the mental health data and behavioral data of social assistance recipients and mining valuable information and patterns from them to provide a scientific basis for psychological intervention is a work of potential significance. By analyzing the data obtained from the questionnaire survey, the system can reveal the mental health problems and risk factors that the recipients may have and propose corresponding intervention suggestions and measures, as shown in Table 1.

**Table 1:** Psychological intervention strategies for social assistance recipients.

Different dimensions	Specific measures
Personalized psychological intervention plans	For those recipients who engage frequently in social networking activities but experience significant emotional fluctuations, training courses on emotional regulation and stress management can be provided to help them better cope with stress and emotional fluctuations.
Provision of psychological counseling services	A platform can be established for psychological counseling services, allowing recipients to access professional psychological counseling support anytime, anywhere. Through analysis of social networking behavior data, recipients who may need psychological counseling support can be identified and timely assistance can be provided. Psychological counseling services can include various forms such as online chat, telephone counseling, and face-to-face counseling to meet the needs of different recipients.
Establishment of support networks	By analyzing social networking behavior data, the recipients’ social relationship networks can be identified to establish support networks, allowing recipients to feel more social and emotional support. Support networks can include different groups such as family members, friends, and colleagues, who can provide support and encouragement when recipients face difficulties or challenges.
Regular tracking and evaluation	A tracking mechanism can be established to regularly assess and monitor the mental health status of recipients. Through continuous data collection and analysis, changes and trends in mental health problems can be promptly identified, and intervention measures can be adjusted accordingly.
Provision of mental health education	Social networking platforms and other channels can be utilized to provide mental health education to recipients, enhancing their awareness and understanding of mental health and improving their mental health literacy. Mental health education can include skills in emotion management, stress relief methods, and interpersonal relationship handling, helping recipients establish positive and healthy psychological behavior habits.
Community support and participation	Community psychological health support groups can be established, allowing recipients to receive more support and assistance within the community. Through analysis of social networking data, recipients who may be suitable for participation in support groups can be identified and invited to join. Community support groups can provide emotional support, information support, and practical assistance, helping recipients better cope with challenges and difficulties in life.

Participants generally express a desire for the system to be optimized and improved in areas such as personalized recommendations, ease of use, and treatment effectiveness. These feedbacks provide clear direction and goals for future system enhancements. The above intervention suggestions and measures aim to provide scientific psychological intervention support to social assistance recipients by analyzing psychological health data and behavioral data. The goal is to help them improve their mental health status, enhance their quality of life, and achieve self-development and growth. Meanwhile, these measures also require collaboration with professional mental health institutions and professionals to jointly provide comprehensive mental health services to the recipients.

## 5 Conclusion

The survey conducted reveals that social assistance recipients generally express satisfaction or high satisfaction with the IMHAS. They believe that the system effectively improves their mental health status and provides personalized intervention plans. This indicates that the system has played an important role in social assistance, contributing to the enhancement of recipients’ mental health levels. Respondents generally believe that the personalized intervention plans provided by the system are in line with their actual situations and needs. This demonstrates the significant importance of personalized intervention in mental health assistance, as it can better meet the needs of recipients and improve intervention effectiveness. Participants show varying degrees of satisfaction with the system’s personalized recommendations, types of interventions, delivery methods, and treatment outcomes. Among these, personalized recommendations and treatment effectiveness are the two areas that participants are most concerned with.

A rigorous data governance framework is established in this study, including data encryption, anonymization and access control. All data is encrypted with the Advanced Encryption Standard during collection, storage and processing to ensure security in data transmission and storage. To enhance the transparency and interpretability of the algorithm, the development process of the AI system, as well as the algorithms and model architectures adopted, are documented in detail. A Data Security Emergency Response Plan is also formulated: in the event of a data breach, affected users would be notified within 24 h, with details on the scope of the breach, risk level and remedial measures. Meanwhile, the incident would be reported to the ethics review committee and data protection regulatory authorities, and corresponding liabilities would be assumed. Multiple channels are used to recruit participants, including communities, medical institutions and online platforms, to ensure the diversity and representativeness of the sample. However, the principle of voluntary participation may lead to self-selection bias in the sample, i.e., individuals who pay more attention to mental health issues or are more willing to accept new technological interventions may be more inclined to participate in the study. Such bias may affect the generalizability and applicability of the research results. To mitigate this bias, random sampling methods will be considered in subsequent studies, and efforts will be made to expand the recruitment scope to attract more participants with diverse backgrounds and needs. At the same time, sensitivity analysis will be conducted on the research results to evaluate the impact of different sample characteristics on the results, thereby improving the robustness of the research conclusions.

Although this study reports the significant effectiveness of the IMHAS system in improving the mental health of social assistance beneficiaries, a cross-sectional research design with limited longitudinal follow-up is adopted. This design restricts the evaluation of the long-term sustainability of intervention effects and the accurate inference of causal relationships. Future research should consider adopting a longitudinal follow-up design with a longer duration and even conducting randomized controlled trials to more accurately assess the sustainability of intervention effects and causal relationships. In addition, as participants took part on a voluntary basis, individuals who pay more attention to mental health issues or are more willing to accept new technological interventions may be more inclined to participate in the study. Such self-selection bias may affect the generalizability and applicability of the research results. Future research should strive to expand the recruitment scope and adopt more random sampling methods to reduce selection bias. This study relies on self-reported measurement tools to assess mental health status. Although these tools are widely used and have certain validity in mental health research, they may still be affected by participants’ subjective bias. Future research should consider combining objective physiological indicators and more comprehensive assessment tools to improve the accuracy of measurements.

## Data Availability

The original contributions presented in the study are included in the article/supplementary material, further inquiries can be directed to the corresponding author.

## References

[ref1] Alam A. (2022). Investigating sustainable education and positive psychology interventions in schools towards achievement of sustainable happiness and wellbeing for 21st century pedagogy and curriculum. ECS Trans. 107:19481. doi: 10.1149/10701.19481ecst

[ref2] Alowais S. Alghamdi S. Alsuhebany N. Alqahtani T. Alshaya A. Almohareb S. . (2023). Revolutionizing healthcare: the role of artificial intelligence in clinical practice. BMC Med. Educ. 23:689. doi: 10.1186/s12909-023-04698-z, 37740191 PMC10517477

[ref3] Barbalat G. Plasse J. Chéreau-Boudet I. Gouache B. Legros-Lafarge E. Massoubre C. . (2024). Contribution of socio-demographic and clinical characteristics to predict initial referrals to psychosocial interventions in patients with serious mental illness. Epidemiol. Psychiatr. Sci. 33:e2. doi: 10.1017/S2045796024000015, 38282331 PMC10894705

[ref4] Boyer P. (2022). Why we blame victims, accuse witches, invent taboos, and invoke spirits: a model of strategic responses to misfortune. J. R. Anthropol. Inst. 28, 1345–1364. doi: 10.1111/1467-9655.13826

[ref5] Chen Z. H. Lin L. Wu C. F. Li C. F. Xu R. H. Sun Y. (2021). Artificial intelligence for assisting cancer diagnosis and treatment in the era of precision medicine. Cancer Commun. 41, 1100–1115. doi: 10.1002/cac2.12215, 34613667 PMC8626610

[ref6] Doraiswamy P. M. Blease C. Bodner K. (2020). Artificial intelligence and the future of psychiatry: insights from a global physician survey. Artif. Intell. Med. 102:101753. doi: 10.1016/j.artmed.2019.101753, 31980092

[ref7] Fei Z. Yang E. Li D. D.-U. Butler S. Ijomah W. Li X. . (2020). Deep convolution network based emotion analysis towards mental health care. Neurocomputing 388, 212–227. doi: 10.1016/j.neucom.2020.01.034

[ref8] Fu Z. Burger H. Arjadi R. Bockting C. L. H. (2020). Effectiveness of digital psychological interventions for mental health problems in low-income and middle-income countries: a systematic review and meta-analysis. Lancet Psychiatry 7, 851–864. doi: 10.1016/s2215-0366(20)30256-x, 32866459 PMC7455253

[ref9] Goldberg S. B. Flemotomos N. Martinez V. R. Tanana M. J. Kuo P. B. Pace B. T. . (2020). Machine learning and natural language processing in psychotherapy research: Alliance as example use case. J. Couns. Psychol. 67, 438–448. doi: 10.1037/cou0000382, 32614225 PMC7393999

[ref10] Gual-Montolio P. Jaén I. Martínez-Borba V. Castilla D. Suso-Ribera C. (2022). Using artificial intelligence to enhance ongoing psychological interventions for emotional problems in real- or close to real-time: a systematic review. Int. J. Environ. Res. Public Health 19:7737. doi: 10.3390/ijerph19137737, 35805395 PMC9266240

[ref11] Hou T. Zhang T. Cai W. Song X. Chen A. Deng G. . (2020). Social support and mental health among health care workers during coronavirus disease 2019 outbreak: a moderated mediation model. PLoS One 15:e0233831. doi: 10.1371/journal.pone.0233831, 32470007 PMC7259684

[ref12] Jolly P. Kong D. T. Kim K. Y. (2020). Social support at work: an integrative review. J. Organ. Behav. 42, 229–251. doi: 10.1002/job.2485

[ref13] Le Glaz A. Haralambous Y. Kim-Dufor D. H. Lenca P. Billot R. Ryan T. C. . (2021). Machine learning and natural language processing in mental health: systematic review. J. Med. Internet Res. 23:e15708. doi: 10.2196/15708 33944788, 33944788 PMC8132982

[ref14] Lee D. Yoon S. N. (2021). Application of artificial intelligence-based Technologies in the Healthcare Industry: opportunities and challenges. Int. J. Environ. Res. Public Health 18:271. doi: 10.3390/ijerph18010271, 33401373 PMC7795119

[ref15] Leung Y. W. Wouterloot E. Adikari A. Hirst G. de Silva D. Wong J. . (2021). Natural language processing-based virtual cofacilitator for online cancer support groups: protocol for an algorithm development and validation study. JMIR Res. Protoc. 10:e21453. doi: 10.2196/21453, 33410754 PMC7819785

[ref16] Mishra S. (2020). Social networks, social capital, social support and academic success in higher education: a systematic review with a special focus on ‘underrepresented’ students. Educ. Res. Rev. 29:100307. doi: 10.1016/j.edurev.2019.100307

[ref17] Mitchell E. (2019). Negotiating vulnerability: the experience of long-term social security recipients. Sociol. Rev. 68:003802611987677. doi: 10.1177/0038026119876775

[ref18] Mylona A. Avdi E. Paraskevopoulos E. (2022). Alliance rupture and repair processes in psychoanalytic psychotherapy: multimodal in-session shifts from momentary failure to repair. Couns. Psychol. Q. 35, 814–841. doi: 10.1080/09515070.2021.2013162

[ref19] Parker S. Grote G. (2019). Automation, algorithms, and beyond: why work design matters more than ever in a digital world. Appl. Psychol. 71, 1171–1204. doi: 10.1111/apps.12241

[ref20] Qiu M. Wu Y. (2024). Understanding the experience of family caregivers of patients with leukemia: a qualitative analysis of online blogs. Humanit. Soc. Sci. Commun. 11, 1–11. doi: 10.1057/s41599-024-02830-y

[ref21] Rowe J. P. Lester J. C. (2020). Artificial intelligence for personalized preventive adolescent healthcare. J. Adolesc. Health 67, S52–S58. doi: 10.1016/j.jadohealth.2020.02.021 32718516

[ref22] Saiyu G. Shali W. Yanfang L. Jingping Z. (2022). Mental health of recipients after cadaveric liver transplantation: a perspective from positive psychology. J. Clin. Nurs. 32, 4710–4718. doi: 10.1111/jocn.16530, 36320122

[ref23] Sanderson W. C. Arunagiri V. Funk A. P. Ginsburg K. L. Krychiw J. K. Limowski A. R. . (2020). The nature and treatment of pandemic-related psychological distress. J. Contemp. Psychother. 50, 251–263. doi: 10.1007/s10879-020-09463-7, 32836377 PMC7320243

[ref24] Shao R. He P. Ling B. Tan L. Xu L. Hou Y. . (2020). Prevalence of depression and anxiety and correlations between depression, anxiety, family functioning, social support and coping styles among Chinese medical students. BMC Psychol. 8:38. doi: 10.1186/s40359-020-00402-8, 32321593 PMC7178943

[ref25] Sloshower J. Guss J. Krause R. Wallace R. Williams M. Reed S. . (2019). Psilocybin-assisted therapy of major depressive disorder using acceptance and commitment therapy as a therapeutic frame. J. Context. Behav. Sci. 15, 12–19. doi: 10.1016/j.jcbs.2019.11.002

[ref26] Sun W. Bocchini P. Davison B. D. (2020). Applications of artificial intelligence for disaster management. Nat. Hazards 103, 2631–2689. doi: 10.1007/s11069-020-04124-3

[ref27] Torous J. Bucci S. Bell I. H. Kessing L. V. Faurholt-Jepsen M. Whelan P. . (2021). The growing field of digital psychiatry: current evidence and the future of apps, social media, chatbots, and virtual reality. World Psych. 20, 318–335. doi: 10.1002/wps.20883, 34505369 PMC8429349

[ref28] Xu L. Sanders L. Li K. Chow J. C. L. (2021). Chatbot for health care and oncology applications using artificial intelligence and machine learning: systematic review. JMIR Cancer 7:e27850. doi: 10.2196/27850, 34847056 PMC8669585

[ref29] Zhang M. Ding H. Naumceska M. Zhang Y. (2022). Virtual reality technology as an educational and intervention tool for children with autism Spectrum disorder: current perspectives and future directions. Behav. Sci. 12:138. doi: 10.3390/bs12050138, 35621435 PMC9137951

[ref30] Zhang J. Wu W. Zhao X. Zhang W. (2020). Recommended psychological crisis intervention response to the 2019 novel coronavirus pneumonia outbreak in China: a model of West China hospital. Precision Clin. Med. 3, 3–8. doi: 10.1093/pcmedi/pbaa006, 35960676 PMC7107095

